# X-ray diffraction pattern from the flight muscle of *Toxorhynchites towadensis* reveals the specific phylogenic position of mosquito among Diptera

**DOI:** 10.1186/s40851-015-0024-1

**Published:** 2015-08-11

**Authors:** Hiroyuki Iwamoto

**Affiliations:** Japan Synchrotron Radiation Research Institute, SPring-8, 1-1-1 Kouto, Sayo-cho, Sayo-gun, Hyogo 679-5198 Japan

**Keywords:** Mosquito, Insect flight muscle, Synchrotron radiation, X-ray diffraction

## Abstract

**Introduction:**

The Diptera are a group of insects with only a single pair of wings (forewings), and are considered monophyletic (originating from a common ancestor). The flight muscle in Diptera has features not observed in other insects, such as the long Pro-Ala-rich peptide associated with tropomyosin, not with troponin-I as in other insects, and the formation of a superlattice by myosin filaments analogous to that in vertebrate skeletal muscle.

**Results:**

Here we describe X-ray diffraction patterns from the flight muscle of a mosquito, *Toxorhynchites towadensis* (Culicidae), belonging to a primitive group of Diptera. The diffraction pattern indicates that myosin filaments in the flight muscle of this species do not form a superlattice. X-ray diffraction also shows meridional reflections that are not observed in other dipterans, but are present in the patterns from bumblebee (Hymenoptera) flight muscle.

**Conclusion:**

These observations suggest that the superlattice structure evolved after the common ancestor of Diptera had diverged from other insects. The flight muscle of mosquito may retain primitive structural features that are shared by Hymenoptera.

## Introduction

Mosquitos belong to the insect order Diptera, which is characterized by having only one pair of wings. Diptera is one of the orders of insects that possess asynchronous flight muscles (asynchronous IFM), capable of driving their wings at high frequencies not achievable by the repetition of ordinary contraction-relaxation cycles. The reported frequencies of wing beat of mosquitos range between 320 and 800 Hz [1–7]. These previous studies have shown that the buzzing sound produced in this frequency range is also utilized for male-female communication/species recognition.

**Fig. 1:**
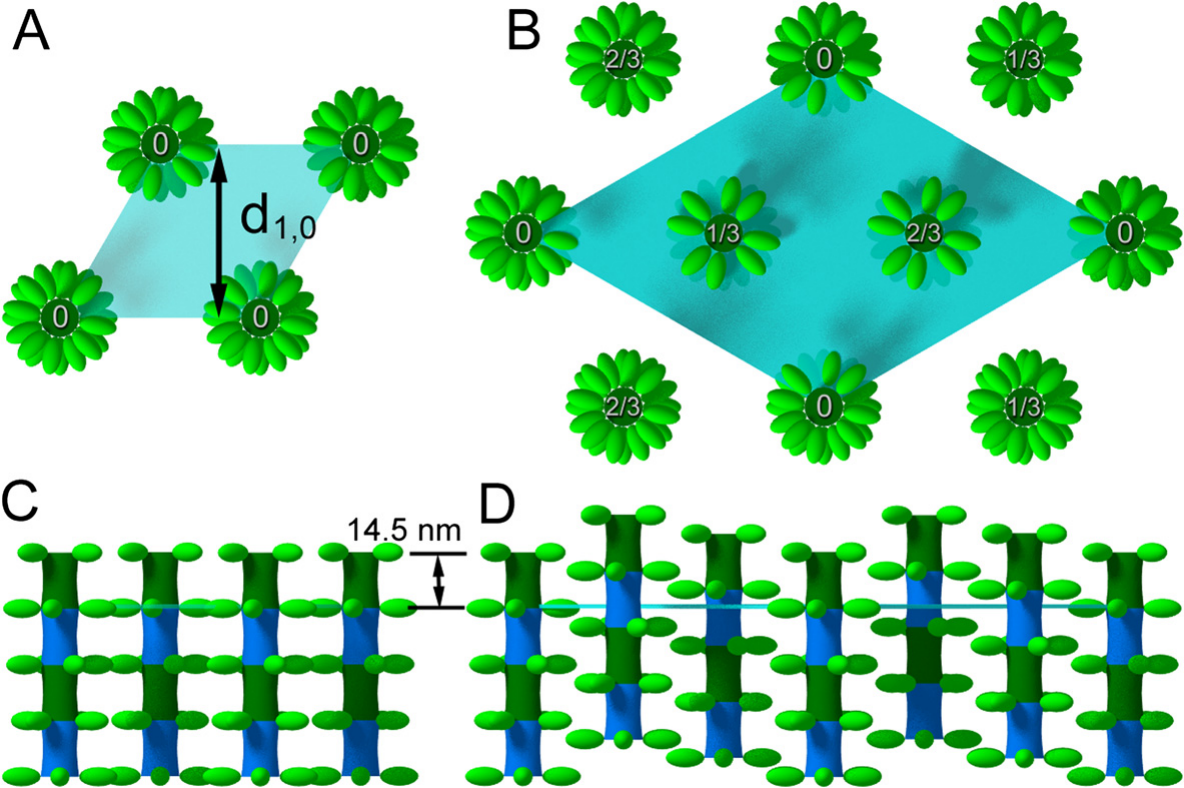
Simple lattice and superlattice structures of myosin filaments in insect flight muscle. **a**, **c** simple lattice seen in insect orders other than Diptera; **b**, **d** superlattice seen in higher dipterans. **a**, **b** top view; **c**, **d** side view. Based on [12]

Although asynchronous IFM is believed to have occurred many times independently in the course of insect evolution, Diptera is considered to be monophyletic [8]. The asynchronous IFM of dipterans is known to have features not observed in other insects. One is the flight muscle-specific tropomyosin, which has a long proline-alanine-rich sequence at its C-terminus [9–11]. This sequence is observed in the flight muscles of all winged insects, but it is associated with troponin-I in other orders [9]. A second characteristic feature is the superlattice arrangement of the thick filaments [12]. In this arrangement, the position of the crown of myosin heads is axially shifted by one third of the 14.5-nm basic repeat with respect to its neighboring thick filaments (Fig. 1). This shift is not only inferred from X-ray diffraction, but is also confirmed by the analysis of electron micrographs (12). As a result, the thick filaments in a sarcomere form a lattice that has a larger unit cell size than that of a simple lattice in which all the thick filaments have the same axial position. Lattice structure is readily identified by recording X-ray diffraction patterns; in the case of the simple lattice, the intensity of the 1st myosin meridional reflection has a single peak on the meridian, whereas in the case of the superlattice, the intensity forms two peaks. This was first observed in *Drosophila* flight muscle [12], but was later confirmed in other species ([13] as well as in the present study).

Mosquitos (Culicidae) are considered to be one of the most primitive groups of dipterans; it is thus of interest to know whether they also exhibit these features. However, their body sizes are generally small, and it is difficult to prepare flight muscle specimens suitable for recording high-quality X-ray diffraction patterns. Here, we describe the diffraction patterns from the IFM of the largest mosquito species in Japan, *Toxorhynchites towadensis* (elephant mosquito). The length of its dorsal longitudinal muscle fibers exceeds 2 mm, which makes it possible to use the mounting technique used for larger insects. The high-quality diffraction patterns recorded in this way revealed many features that are not observed in other (higher) dipteran species.

## Materials and methods

### Materials

**Fig. 2:**
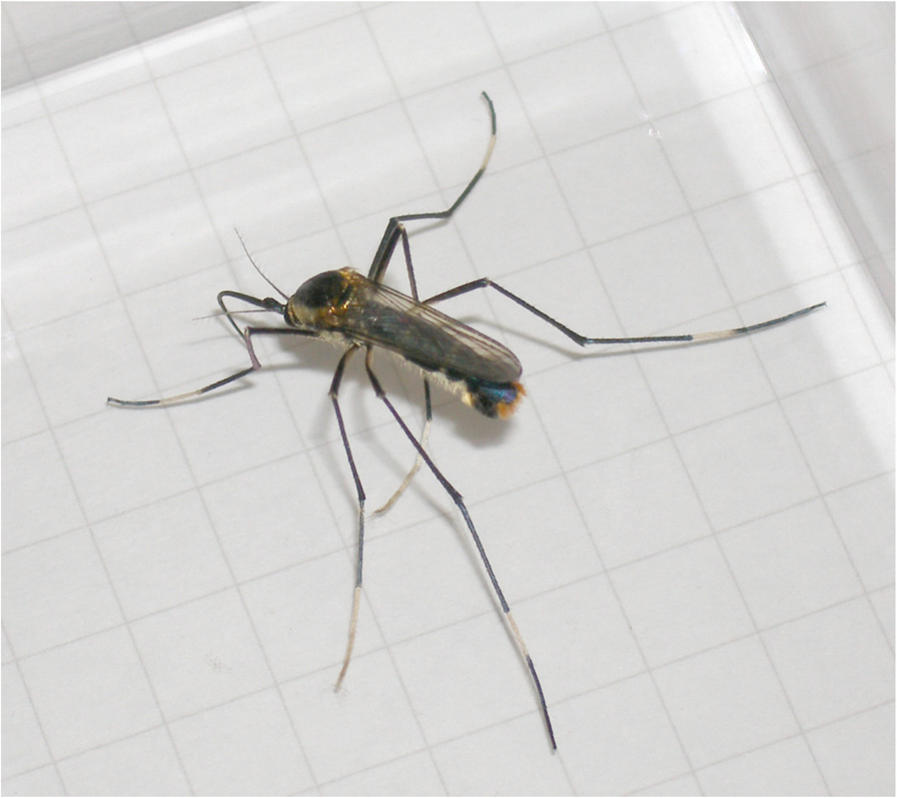
Picture of the giant mosquito (*Toxorhynchites towadensis*) that was used for the X-ray experiment. The size of the mesh is 5 mm

Live mosquitoes (*Toxorhynchites towadensis*) were collected in a building close to the campus of the SPring-8 synchrotron facility in Harima, Japan (Fig. 2). This species was readily identified by its size, bluish metallic body color, and curved proboscis. Its IFM was glycerinated in a 50 % mixture of glycerol and a relaxing solution with protease inhibitors, and stored at –20 °C, as previously described [13]. On the day before the experiment, the dorsal longitudinal muscle fibers were mounted to the specimen chamber by clamping both ends with half-split gold meshes for electron microscopy [13–15]. The composition of solutions (relaxing and rigor) was as described previously [16, 17]. For comparison, flight muscles from other dipterans and bumblebee (*Bombus ignitus*) were also used; these were prepared in the same manner. These dipterans include *Phytomia zonata* (hover fly), *Promachus yesonicus* (robber fly), *Tipula coquilletti* (crane fly) and *Bibio* sp. (march fly), and all these specimens were collected in or near the campus of SPring-8.

### X-ray diffraction recording

X-ray diffraction patterns were recorded at the BL45XU beamline of SPring-8 [18]. The specimen-to-detector distance was 2.4 m, and the X-ray wavelength was 0.1 nm. As a detector, a cooled CCD camera (C4880-50, Hamamatsu Photonics, Hamamatsu, Japan; 1000 × 1018 pixels, pixel size, 150 × 150 μm) was used in combination with an image intensifier (VP5445-MOD, Hamamatsu Photonics). Some of the patterns from bumblebee muscle fibers were recorded on Imaging Plates (Fuji Film Co., Tokyo, Japan) with a pixel size of 50 × 50 μm. The diffraction patterns recorded under the same experimental condition were summed to improve the signal-to-noise ratio, and the background scattering was subtracted by the method described previously [15, 19].

## Results

**Fig. 3:**
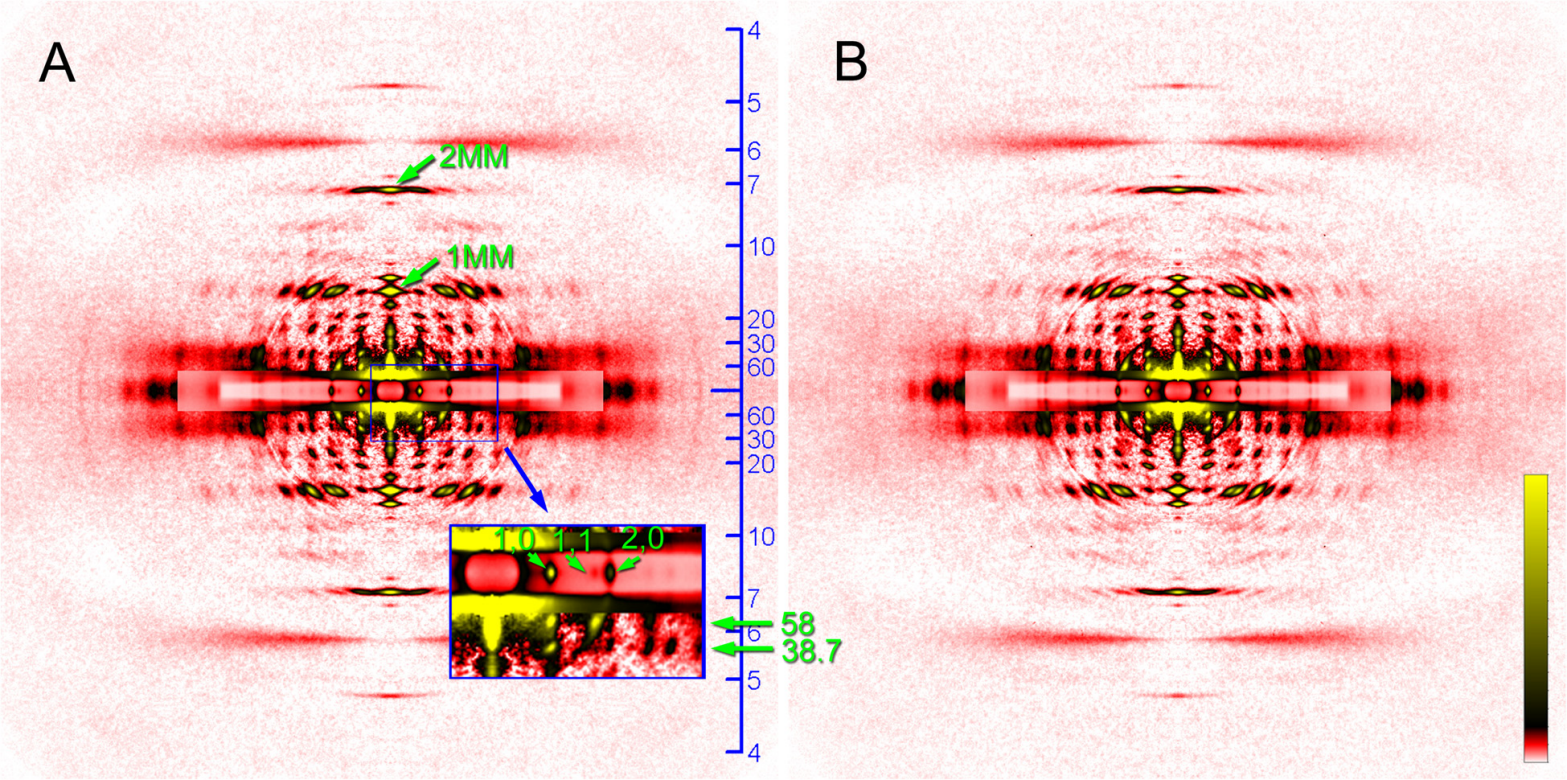
Diffraction patterns recorded from the flight muscle of *Toxorhynchites towadensis*. **a** relaxed; **b** rigor. The low-angle area of the diffraction pattern in (**a**) is magnified in the box on the lower right. In the box, the innermost equatorial reflections (1,0, 1,1 and 2,0) and the two lowest-angle layer-line reflections (*d*-spacing, 58 and 38.7 nm) are indicated by green arrows. Note that the 1st and 2nd myosin meridional reflections (1MM and 2MM) have single peaks right on the meridian. The scale on the right edge of panel **a** indicates *d*-spacing in nm

Diffraction patterns recorded from the mosquito IFM fibers are shown in Fig. 3. Like IFM diffraction patterns from other insects, the diffraction pattern from the mosquito IFM consists of a number of layer-line reflections (ladder-rung-like reflections originating from the helical arrangement of proteins along the filament axis) and meridional reflections (usually spot-like reflections that appear right on the meridian, originating from the axial repeat of monomers of myofilaments). Most of these reflections are indexable to the 38.7-nm long-pitch helical repeat of the actin filament with its associated regulatory proteins, and the 4-start helix of the myosin filament (116 nm basic repeat and 14.5-nm monomer pitch). These features have been described previously in detail for the IFM from the giant waterbug, *Lethocerus* [20]. A number of equatorial reflections, originating from the hexagonal arrangement of myofilaments within a sarcomere, are also observed. The intense 2,0 reflection and the much weaker 1,1 reflection indicate that the hexagonal lattice structure is such that a thin filament is positioned midway between the two neighboring thick filaments, like in other insects (for reasoning see ref. [21]). The unit cell size of the hexagonal lattice (the *d*-spacing for the 1,0 reflection) is ~45 nm, and the value is smaller in rigor (Fig. 3b) than in the relaxed state (Fig. 3a). Otherwise, the difference between the relaxed and rigor patterns is small, except for the slightly stronger actin-based layer line reflections in rigor.

In principle, layer line reflections that arise from the helical arrangement of objects have continuous intensity profiles. If the objects are arranged in a crystalline lattice, the layer lines have intensities only on the spots where the conditions of diffraction are met. This phenomenon is called lattice sampling.

**Fig. 4:**
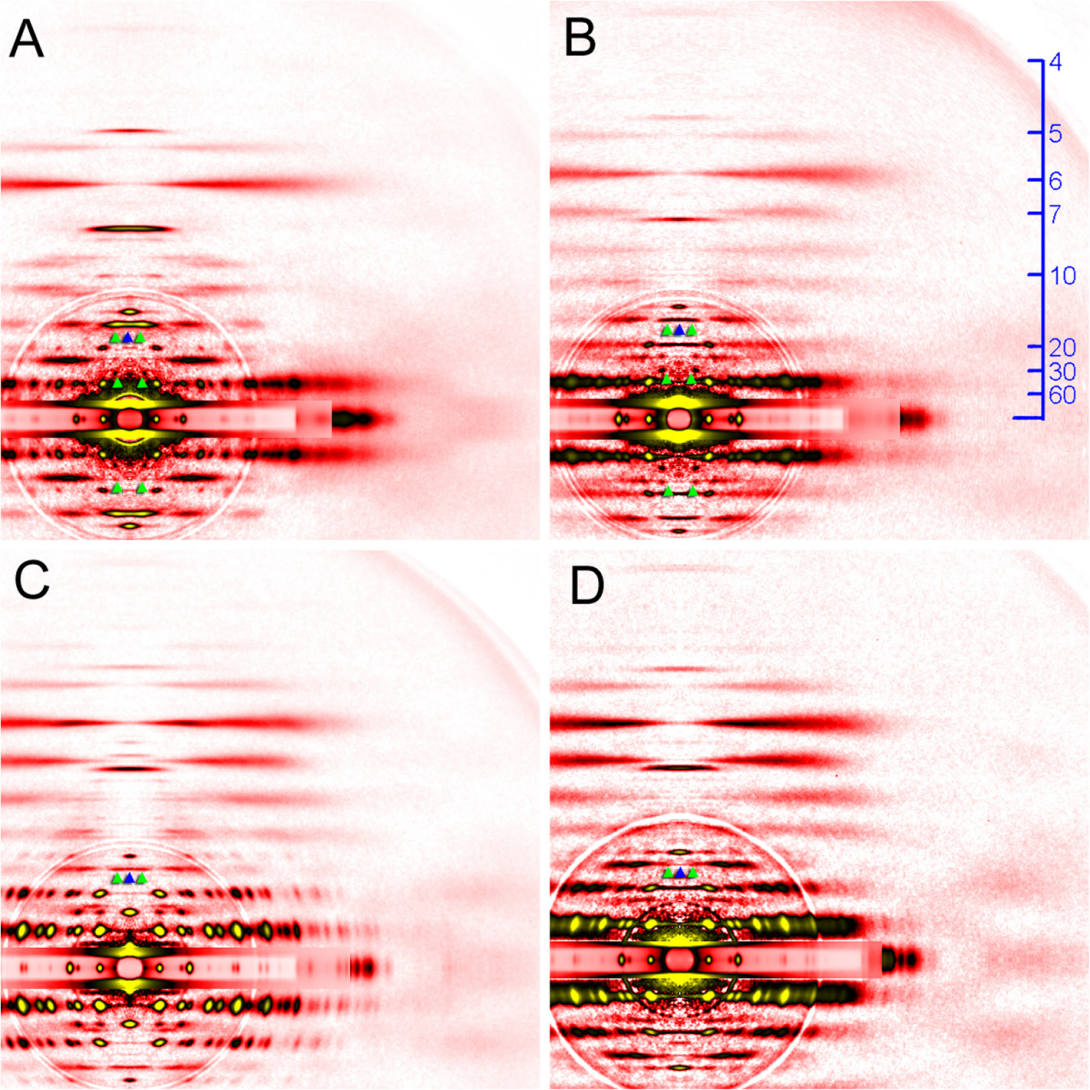
Diffraction patterns from the flight muscles of other dipteran species. **a** *Phytomia zonata* (hover fly); **b** *Promachus yesonicus* (robber fly); **c** *Tipula coquilletti* (crane fly) and **d** *Bibio* sp. (march fly). Green arrowheads indicate peaks indexable to the thick-filament superlattice with a greater lattice constant. Blue arrowheads indicate that the intensity of the 1st myosin meridional reflection (at *d* = 14.5 nm) is weak on the meridian. The pattern in (**a**) was recorded in the relaxed state, and the others were recorded in the rigor state, in which the 1st myosin meridional reflection is weakened

The layer line reflections of the mosquito, especially those in the lower-angle region are finely sampled into discrete spots, indicating that the contractile proteins in the mosquito flight muscle are in crystalline order. This sampling is observed up to the myosin layer line at *d* = 7.2 nm, indicating that the 3-dimensional lattice structure of the mosquito IFM is very regular (in many other insects, the sampling is restricted to lower-order reflections). The pattern of the sampling is identical for all the layer-line reflections, and coincides with the equatorial reflections. This means that the contractile proteins in the mosquito IFM form a simple lattice (orientations of the proteins on the neighboring filaments are the same). This is in marked contrast to the myosin filament lattice of *Drosophila* IFM [12], which forms a superlattice with a greater lattice constant, similar to the superlattice of vertebrate skeletal muscle [22]. The normal insect lattice has a lattice constant (*d*1,0 spacing) of ~45 nm, and the superlattice has a lattice constant of ~78 nm $\left(=45\times \sqrt{3}\right)$. The superlattice structure manifests itself most conspicuously in the myosin meridional reflection at *d* = 14.5 nm, whose intensity is split in the middle and has peaks at *d* ~ 78 nm when measured along the equator. In contrast, the myosin meridional reflection of the mosquito has a sharp peak right on the meridian (Fig. 3), as in non-dipteran insects, such as giant waterbug [20] and bumblebee [13, 15]. So far, all of the studied dipteran species belonging to suborder Brachycera, including *Drosophila* [12], *Phytomia* (hover fly, Fig. 4a), *Tabanus* [23] (horse fly), and *Promachus* (robber fly, Fig. 4b), and suborder Nematocera, including *Ctenacroscelis* [13], *Tipula* (these two are crane flies) and *Bibio* (march fly, Fig. 4d) are found to have the superlattice structure, and the mosquito as presented here is the first dipteran that does not have the superlattice structure.

**Fig. 5:**
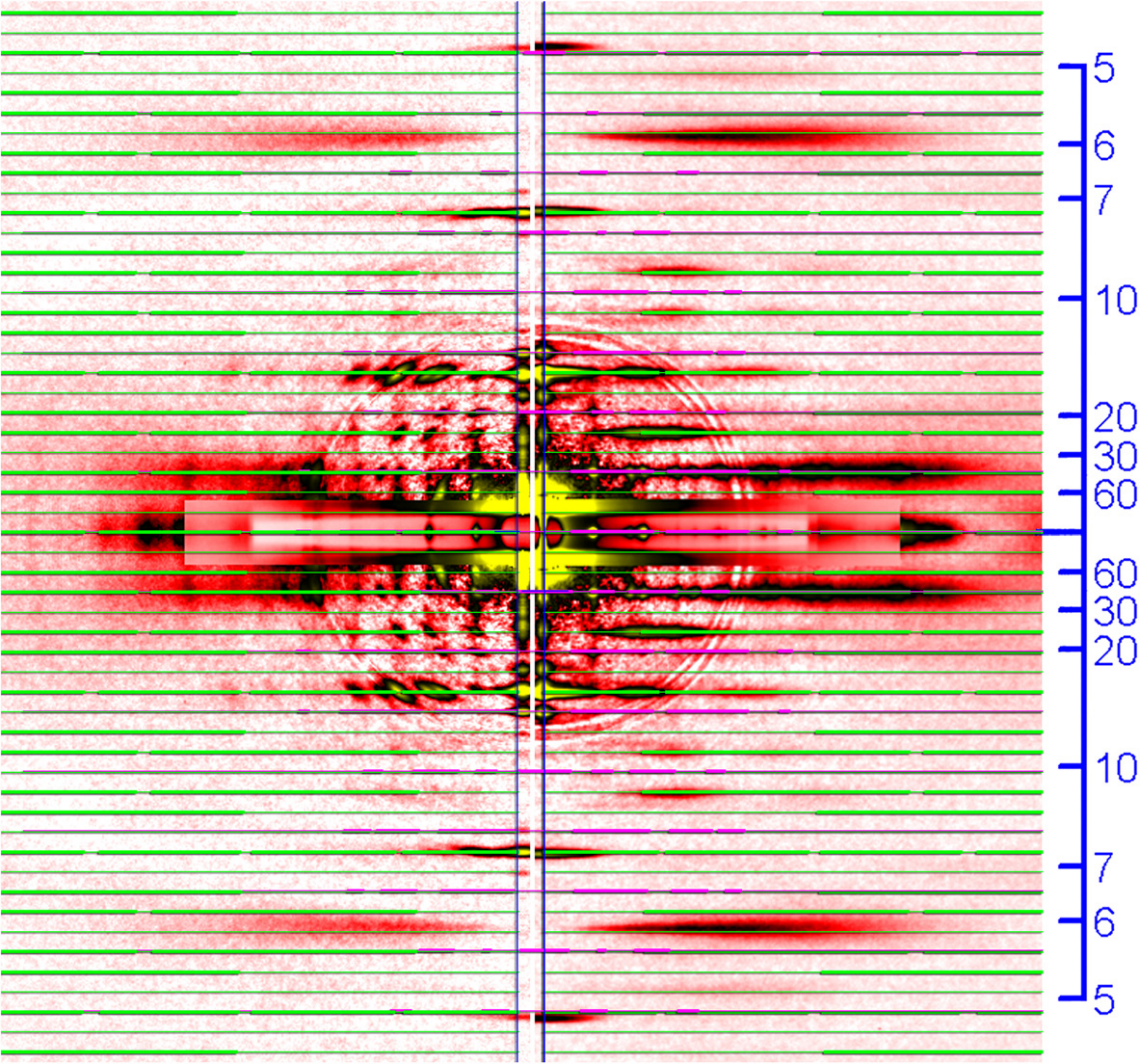
Indexing of reflections in the diffraction pattern from the mosquito (left) and bumblebee (*Bombus*, right) flight muscles. The green lines indicate the positions of reflections expected from the myosin repeat, and the pink lines indicate those expected from the actin repeat. Note the resemblance of positions of meridional reflections that appear at angles lower than 14.5 nm^−1^

The layer line reflections of the mosquito IFM, as shown in Fig. 3, are indexable to either the myosin helix with a basic repeat of 116 nm or the actin helix with a basic repeat of 38.7 nm (Fig. 5). Therefore, the mosquito myofilaments are believed to have the same helical symmetries as those of other insects. Usually, the layer line at *d* = 38.7 nm (combined actin 1st and myosin 3rd (based on the 116-nm basic repeat) is the strongest. The mosquito pattern is unusual in that the 2nd myosin layer line (at *d* = 58 nm) is very intense. This layer line is usually weak even if present, and is clearly recognized only in a few cases (e.g., in giant waterbug [24]).

**Fig. 6:**
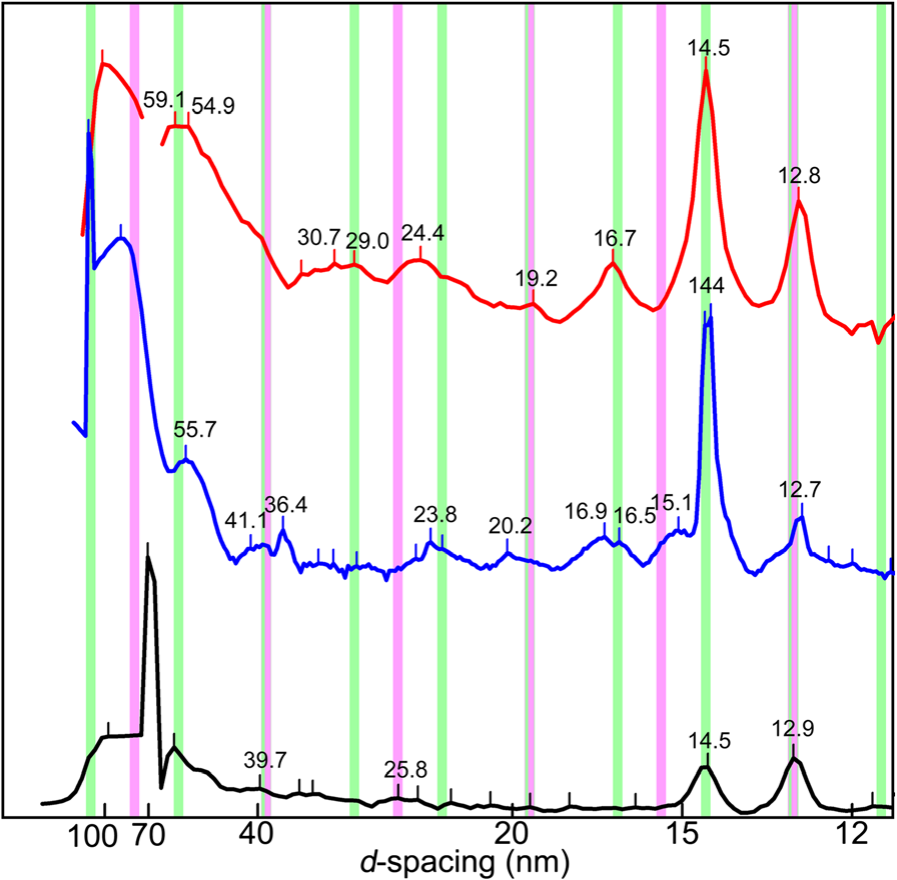
Comparison of intensity profiles of meridional reflections from the flight muscles from the mosquito and other insects. From the top, mosquito, bumblebee (*Bombus*) and hover fly (*Phytomia*). The green lines indicate the positions of reflections expected from the myosin repeat, and the pink lines indicate those expected from the actin repeat. Note that in both mosquito and bumblebee, there are a number of reflections that are not indexable to either myosin or actin repeat. The bumblebee data were recorded by using an Imaging Plate with a pixel size of 50 μm, while others were taken by using a cooled CCD camera with a pixel size of 150 μm. The intensity is normalized with respect to that of the 6th actin layer line at *d* = 5.9 nm

The meridional reflections of the mosquito IFM is very complex when compared with those of other insects (Fig. 5). In the giant waterbug and most of the dipterans (Fig. 4), the meridional reflections are ascribed to either the myosin axial repeat (14.5, 7.25, 4.83 nm, etc.) or the troponin axial repeat (12.9, 6.45, 4.3 nm, etc.). The actual axial troponin repeat is 38.7 nm, but because of its helical arrangement on the six thin filaments surrounding each thick filament, only reflections of the orders of multiples of three have intensities [20]. Therefore, the first meridional reflection that appears in the low-angle region is the 1st-order myosin reflection (8th of the 116-nm repeat) at *d* = 14.5 nm. However, many more reflections are observed on the meridian of the mosquito pattern. Many meridional reflections are observed in the region of *d* > 14.5 nm (Figs. 5 and 6), and they are not readily indexable to known structures. For example, the reflection at *d* = 16.7 nm may be indexed as the 7th of the 116-nm myosin repeat, but an intensity on the meridian is not expected from the 4-start helical symmetry of the myosin filament. The reflection at 24.4 nm is not indexable to any of the known repeats. This situation is similar to the bumblebee flight muscle, which also have many meridional reflections at *d* > 14.5 nm (Figs. 5 and 6). Originally, some of these lower-angle reflections in the bumblebee pattern were indexed to a 155 nm repeat (4 x actin’s 38.7-nm repeat), and were argued that they were of the thin-filament origin. However, higher-resolution recording using Imaging Plates (Fig. 6) reveal that the spacing of these reflections does not exactly coincide with that predicted from the actin repeat. The patterns of occurrence of meridional reflections are similar in mosquito and bumblebee, although they are not identical.

These observations suggest that the structure of the IFM of this primitive dipteran has a higher crystalline order, and is more complex than that of higher dipterans in that it contains more periodically arranged components.

## Discussion

In this study, X-ray diffraction patterns from the flight muscle of a mosquito (*Toxorhynchites towadensis*), one of the most primitive dipterans, were described and compared with those of higher dipterans. It has been shown that the patterns from the mosquito are distinct from those from higher dipterans in that they have reflections that are not found or weak in other dipterans (especially in meridional reflections), and most importantly, they lack a myosin filament superlattice structure.

Dipterans have traditionally been classified into two major groups, the more primitive Nematocera (slender-bodied dipterans with long antennae) and Brachycera (stout flies with short antennae). A recent extensive study of the phylogenetic relationship of dipterans based on morphological and genomic traits [25] places a few groups of former Nematocera, including Culicomorpha (mosquitoes and allies), Psychodomorpha (sand flies and allies) and Tipulomorpha (crane flies and allies) as truly primitive groups of dipterans, while placing Bibionomorpha (march flies and allies) as a sister group of Brachycera. In support of this, the present study has shown that the diffraction patterns from a march fly, *Bibio* have traits similar to those of Brachycera species, i.e., the superlattice structure of the thick filaments and simple meridional reflections (Fig. 4). However, the crane flies (*Ctenacroscelis* and *Tipula*) are found to have these traits, suggesting a closer relationship between Tipulomorpha and Brachycera. To establish that the lack of the superlattice structure and the more complex set of meridional reflections are the primitive traits of dipteran IFM, diffraction patterns should be taken from other primitive families, such as Chilonomidae (midges) and Psychodidae (moth flies); however, these do not include species large enough to guarantee high-quality diffraction patterns. Finally, it is desirable to record diffraction patterns from dipterans belonging to the most primitive Deuterophlebiidae, located at the very base of the dipteran diversification. Again, there is little hope of obtaining diffraction patterns from these, because only one very tiny species (body length, ~2 mm) is known from Japan and it is endangered. In any event, it is clear from the present study that the superlattice structure of the thick filaments is not a trait present in the common ancestor, but it evolved after diversion of dipterans from other groups on insects.

The origin of the unusual meridional reflections from the mosquito IFM, not indexable to the repeats of either actin or myosin filaments, is currently unknown. In bumblebee IFM, similar meridional reflections are observed, and they are resistant to the digestion of the thin filaments by gelsolin (an F-actin-severing protein), suggesting that they are of the thick-filament origin (Iwamoto, unpublished). Therefore, at least in bumblebee, these may represent the repeat of unknown thick-filament proteins. Gelsolin treatment of mosquito IFM fibers is expected to clarify whether their unusual reflections are also of the thick-filament origin. A candidate for such an unknown protein is flightin, a myosin-binding protein found in *Drosophila* [26] and its periodicity is unknown. However, the unusual meridional reflections are not found in *Drosophila* and other Brachycera species, so that it may follow the basic repeat of myosin (116 nm) or the contrast of density along the filament may be so low so that they do not contribute to intensities of meridional reflections. It is also unclear whether the loss of the unknown thick-filament protein(s) and the acquisition of the superlattice structure are coupled.

## Conclusions

The architecture of the myofilament lattice in the flight muscle of the mosquito, *Toxorhynchites towadensis* (Culicidae), retains features observed in other insect orders but not in higher dipterans (simple myosin-filament lattice and structures that give unusual meridional reflections). Examination of the IFM structures of other primitive dipterans is expected to give more insights about the early molecular evolution of dipterans.

## Abbreviations

CCD: Charge-coupled device
IFM: Insect flight muscle
